# Competing risk analyses of overall survival and cancer-specific survival in patients with combined hepatocellular cholangiocarcinoma after surgery

**DOI:** 10.1186/s12885-019-5398-6

**Published:** 2019-02-27

**Authors:** Chaobin He, Yu Zhang, Zhiyuan Cai, Xiaojun Lin

**Affiliations:** 1 Department of Hepatobiliary and Pancreatic Surgery, State Key Laboratory of Oncology in South China, Collaborative Innovation Center for Cancer Medicine, Sun Yat-sen University Cancer Center, Guangzhou, 510060 China; 2 State Key Laboratory of Ophthalmology, Zhongshan Ophthalmic Center, Sun Yat-sen University, Guangzhou, Guangdong 510060 China

**Keywords:** Combined hepatocellular cholangiocarcinoma, Overall survival, Cancer-specific survival, Nomogram, Prognosis

## Abstract

**Background:**

Our objective was to identify risk factors affecting overall survival (OS) and cancer-specific survival (CSS) and build nomograms to predict survival based on a large population-based cohort.

**Methods:**

Two hundred and thirty patients diagnosed with CHCC between 2004 and 2015 were retrospectively extracted from the Surveillance, Epidemiology, and End Results (SEER) database as a training cohort. In addition, Ninety-nine patients diagnosed with CHCC between 2000 and 2017 were retrospectively extracted from Sun Yat-Sen University Cancer Center (SYSUCC) as an external validation. Nomograms for predicting probability of OS and CSS were established. Performance of the nomograms was measured by concordance index (C-index) and the area under receiver operating characteristic (ROC) curve (AUC).

**Results:**

In training cohort, the 1-, 2 and 3-year OS were 67.7, 46.8 and 37.9%, and the 1-, 2 and 3-year CSS were 73.1, 52.0 and 43.0%, respectively. The established nomograms were well calibrated in both training and validation cohort, with concordance indexes (C-index) of 0.652 and 0.659, respectively for OS prediction; 0.706 and 0.763, respectively for CSS prediction. Nomograms also displayed better discriminatory compared with 8th edition tumor-node-metastasis (TNM) stage system for predicting OS and CSS.

**Conclusion:**

We constructed nomograms to predict OS and CSS based on a relatively large cohort. The established nomograms were well validated and could serve to improve predictions of survival risks and guide management of patients with CHCC after surgery.

## Background

Combined hepatocellular cholangiocarcinoma (CHCC) is a rare primary liver cancer, which is composed of mixed elements of both hepatocellular carcinoma (HCC) and cholangiocarcinoma (ICC) [1] and accounts for only 0.4–14.5% of the primary liver cancer [2]. Regarding the treatment of CHCC, patients can obtain the best chance to the greatest survival benefit from surgery [3]. However, CHCC has worse prognosis compared with HCC or ICC [4]. CHCC was firstly described in 1949 by Allen and Lisa [5]. However, due to the low morbidity of CHCC and the absence of unified diagnostic criterion, the clinical and pathological features of CHCC remain unclear. Moreover, different from HCC for which many preoperative prognostic prediction systems have been established [6–8], the prognostic stage system of patients with CHCC remained unclear, varying considerably from different reports [9–11]. The 8th tumor-node-metastasis (TNM) stage system, although it was the most frequently used stage system, it contained some common prognostic factors, such as tumor size, lymph node (LN) metastasis and distant metastasis. In addition, there was no a TNM stage system which is specially designed for CHCC. It was reported that the differences of clinical features among CHCC, HCC and ICC could lead to the variations of prognostic factors [12, 13]. There were also many factors, such as age, gender and tumor grade, which were shown to have great impact on survival. However, they were not included in the TNM stage system. Therefore, it is necessary to establish a stage system which is specially designed for prognostic prediction in patients with CHCC.

In addition, with the improvement of survival of cancer patients, most of patients with CHCC are faced with advanced ages, which are associated with an increasing high rate of comorbidities. Moreover, ta high risk of competing events, which might contribute to more competing deaths, was observed in patients with CHCC as the age increases [14, 15]. Thus, when prognosis is evaluated, competing risks are worthy of being considered. However, most prognostic analyses only focused on overall survival (OS) and ignored the impact of survival from competing events [8, 9, 16]. Competing risk analysis evaluates the informative nature of censoring and the occurrence rates of a particular event, which is more suitable for prognostic analysis. Misleading conclusions might be obtained due to the failure to recognize the presence of competing risks in survival analysis [17].

The present study was to build nomograms to predict 1-, 2-, and 3-year OS and cancer-specific survival (CSS) of these patients based on the Surveillance, Epidemiology, and End Results (SEER) database. Also, another large cohort of patients with CHCC from China was used to externally validate the established nomograms.

## Methods

### Patients

The study population was identified from SEER database from 2004 to 2015. We focused on cases pathologically confirmed CHCC after surgery [International Classification of Diseases for Oncology, Third Edition (ICD-O-3) site code C22.0 and C22.1; histology code: 8180/3]. In addition, consecutive patients with pathological diagnosis of CHCC after surgery between 2000 and 2017 at the department of Hepatobiliary and Pancreatic Surgery of Sun Yat-Sen University Cancer Center (SYSUCC) were also enrolled in the present study. The exclusion criteria are the same as those described in our previous study [10].

### Data collection

Records for the age at diagnosis, gender, tumor site, tumor grade, tumor size, TNM stage, follow-up information and cause of death were retrospectively retrieved from SEER database and the medical management system of SYSUCC. Survival time was defined as the duration from the date of diagnosis to last follow-up or death due to all causes (OS) or CHCC (CSS).

### Nomogram construction and validation

Nomograms were constructed based on cohort from SEER database and externally validated based on cohort from SYSUCC database. Student’s t test and chi-square test or Fisher’s exact test were used to compare continuous variables and categorical variables, respectively. The Kaplan-Meier curves were analyzed by log-rank tests. Univariate analysis and multivariate analysis were constructed using the Cox regression model and hazard ratio (HR) and the associated 95% confidence interval (CI) for each variable were determined. Clinical and pathological factors were analyzed by the Fine and Grey’s model for their cumulative incidence function (CIF) on cancer-specific mortality and non-cancer-specific mortality. Independent prognostic factors identified in the multivariate analysis were used to build nomograms to predict the 1-, 2- and 3-year OS and CSS rates.

As two important aspects of the performance of the established nomograms, the discrimination and calibration power were evaluated by concordance index (C-index) and calibration curves, respectively [18]. Bootstraps with 1000 resamples were used in the validation of the nomogram. In addition, the area under receiver operating characteristic (ROC) curve (AUC) was used to evaluate the precision of the survival predictions.

R version 3.4.2 software (The R Foundation for Statistical Computing, Vienna, Austria. http://www.r-project.org), along with SPSS version 22 (SPSS Inc., Chicago, IL, USA), was used to conduct statistical analyses. A two tailed *P*-value < 0.05 was considered statistically significant.

## Results

### Patient characteristics

Two hundred and thirty patients with CHCC and another ninety-nine patients with CHCC were retrospectively identified from SEER database as training cohort and SYSUCC database as external validation cohort, respectively in the present study. The baseline characteristics of the training cohort and the validation cohort were shown in Table 1. Among these patients, the mean age was 59.8 years and 49.7 years for the patients in the training cohort and validation cohort, respectively. Most patients were male and had tumor origin from liver in both train and validation cohort. Poor differentiation (130, 56.7%) was the most common tumor grade, while most patients had tumors which were moderately differentiated in the validation cohort. The proportions of patients were comparable between two cohorts in terms of T stage (8th), N stage (8th) and 8th edition TNM stage system.

**Table 1:** The comparison of clinicopathological factors between training cohort and validation cohort

Characteristic		N	Patients		P
			Training cohort	Validation cohort	
Total		329	230	99	
Age (median, years)			60.5 ± 10.41	50.0 ± 11.15	
Gender	Male	234	161	73	0.511
	Female	95	69	26	
Tumor site	Intrahepatic biliary tract	29	16	13	0.089
	Liver	300	214	86	
Tumor grade	Well	8	8	0	0.058
	Moderate	143	92	51	
	Poor	178	130	48	
Tumor size	≤ 5 cm	183	137	46	0.056
	> 5 cm	146	93	53	
T stage (8th)	I	137	93	44	0.203
	II	108	83	25	
	III	62	38	24	
	IV	22	16	6	
LN metastasis	Absent	291	204	87	0.852
	Present	38	26	12	
TNM stage (8th)	I	122	79	43	0.509
	II	104	77	27	
	III	103	74	29	

*LN* lymph node, *TNM* Tumor-Node-Metastasis

### OS and CSS of patients

**Fig. 1:**
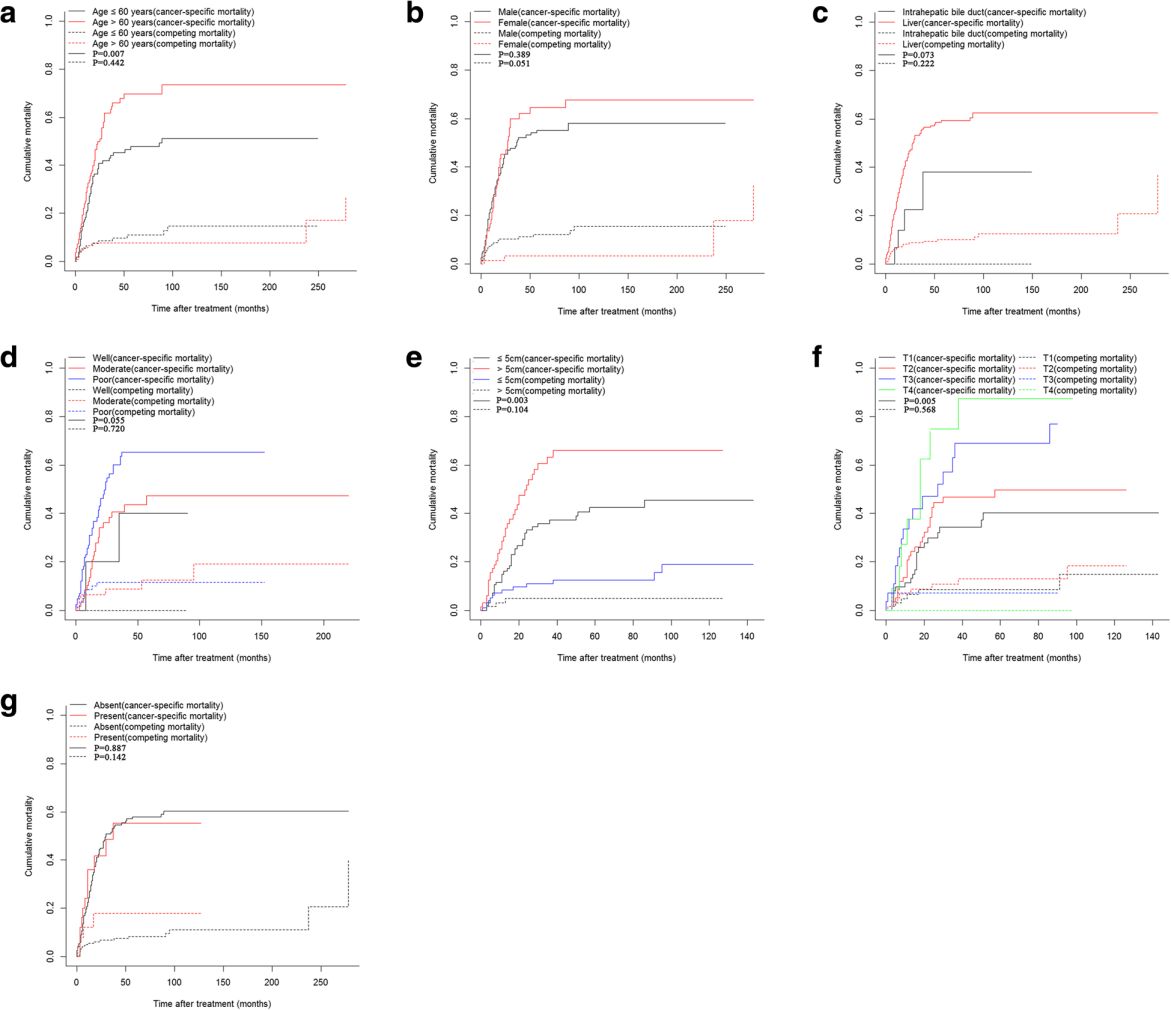
Cumulative cancer-specific and competing mortality according to patient characteristics: (**a**) Age; (**b**) Gender; (**c**) Tumor site; (**d**) Tumor grade; (**e**) Tumor size; (**f**) T stage (8th); (**g**) N stage (8th). Abbreviations: LN, lymph node

During the follow-up period, deaths were observed in 142 out of 230 (61.7%) patients in the training cohort and 43 out of 99 (43.4%) patients in the validation cohort. In the training cohort, CHCC contributed to deaths of 118 (51.3%) patients and competing risk events contributed to deaths of 24 (10.4%) patients. In the validation cohort, there were 31 cancer-specific death and 12 non-cancer-specific death during the follow-up period. Table 2 outlined the comparisons of 1-, 2- and 3-year OS rates, cancer-specific mortalities and non-cancer-specific mortalities of patients. It was shown that older age, larger tumor and advanced T stage (8th) were responsible for higher cumulative rates of cancer-specific-mortality. Earlier N stage (8th), well differentiation and origin from intrahepatic bile duct seemed to be related to the decreased cancer-special-mortalities while the differences were not significant (Fig. 1).

**Table 2:** Overall survival rates and cumulative incidences of mortality among patients with CHCC after surgery

Characteristic		Patients		Overall survival rate (%)			P	Cancer-specific mortality (%)			P	Non-cancer-specific mortality (%)			P
		No.	%	1-year	2-year	3-year		1-year	2-year	3-year		1-year	2-year	3-year	
Total		230	100	67.7	46.8	37.9		25.9	45.2	53.4		5.9	8.0	8.0	
Age (years)	≤ 60	115	50	71.8	50.2	46.9	0.014	21.1	40.8	44.2	0.007	6.4	8.5	8.5	0.442
	> 60	115	50	63.5	42.8	28.4		30.8	49.9	63.2		5.5	7.6	7.6	
Gender	Male	161	70	64.5	44.4	39.3	0.742	27.2	45.2	50.4	0.389	7.9	10.1	10.1	0.051
	Female	69	30	74.9	51.5	35.3		22.9	45.3	60.0		1.5	3.3	3.3	
Tumor site	Intrahepatic biliary tract	16	7	93.3	77.5	77.5	0.019	6.7	22.5	22.5	0.073	0	0	0	0.222
	Liver	214	93	65.8	44.4	35.5		27.4	46.8	55.2		6.4	8.6	8.6	
Tumor grade	Well	8	3	80.0	80.0	60.0	0.016	20.0	20.0	40.0	0.055	0	0	0	0.720
	Moderate	92	40	76.4	55.1	50.7		17.1	36.2	40.6		6.5	8.7	8.7	
	Poor	130	57	59.4	35.5	23.7		31.0	53.2	63.6		8.5	11.5	11.5	
Tumor size	≤ 5 cm	137	60	74.8	55.4	51.2	0.041	16.0	33.1	37.4	0.003	8.4	10.9	10.9	0.104
	> 5 cm	93	40	65.9	43.6	30.3		30.4	51.8	63.3		3.1	4.9	4.9	
T stage (8th)	I	93	40	82.4	62.3	55.7	0.015	11.3	29.9	34.3	0.005	6.6	8.5	8.5	0.568
	II	83	36	69.5	46.2	39.6		44.5	42.4	46.7		6.8	10.6	10.6	
	III	38	17	55.2	45.8	23.8		37.7	47.0	69.1		7.1	7.1	7.1	
	IV	16	7	57.1	22.9	11.4		37.7	75.1	75.1		0	0	0	
LN metastasis	Absent	204	89	70.1	48.4	39.6	0.253	24.2	44.9	53.1	0.887	5.5	6.7	6.7	0.142
	Present	26	11	52.0	40.4	27.0		36.0	41.8	48.5		12.0	17.8	17.8	
TNM stage (8th)	I	79	34	81.5	62.7	55.4	0.014	11.3	32.5	37.4	0.084	5.6	5.6	5.6	0.896
	II	63	27	74.9	53.7	51.0		18.0	39.0	41.6		2.2	7.3	7.3	
	III	88	39	55.2	40.0	27.3		35.7	48.0	60.6		9.2	12.1	12.1	

*CHCC* combined hepatocellular cholangiocarcinoma, *LN* lymph node, *TNM* Tumor-Node-Metastasis

**Fig. 2:**
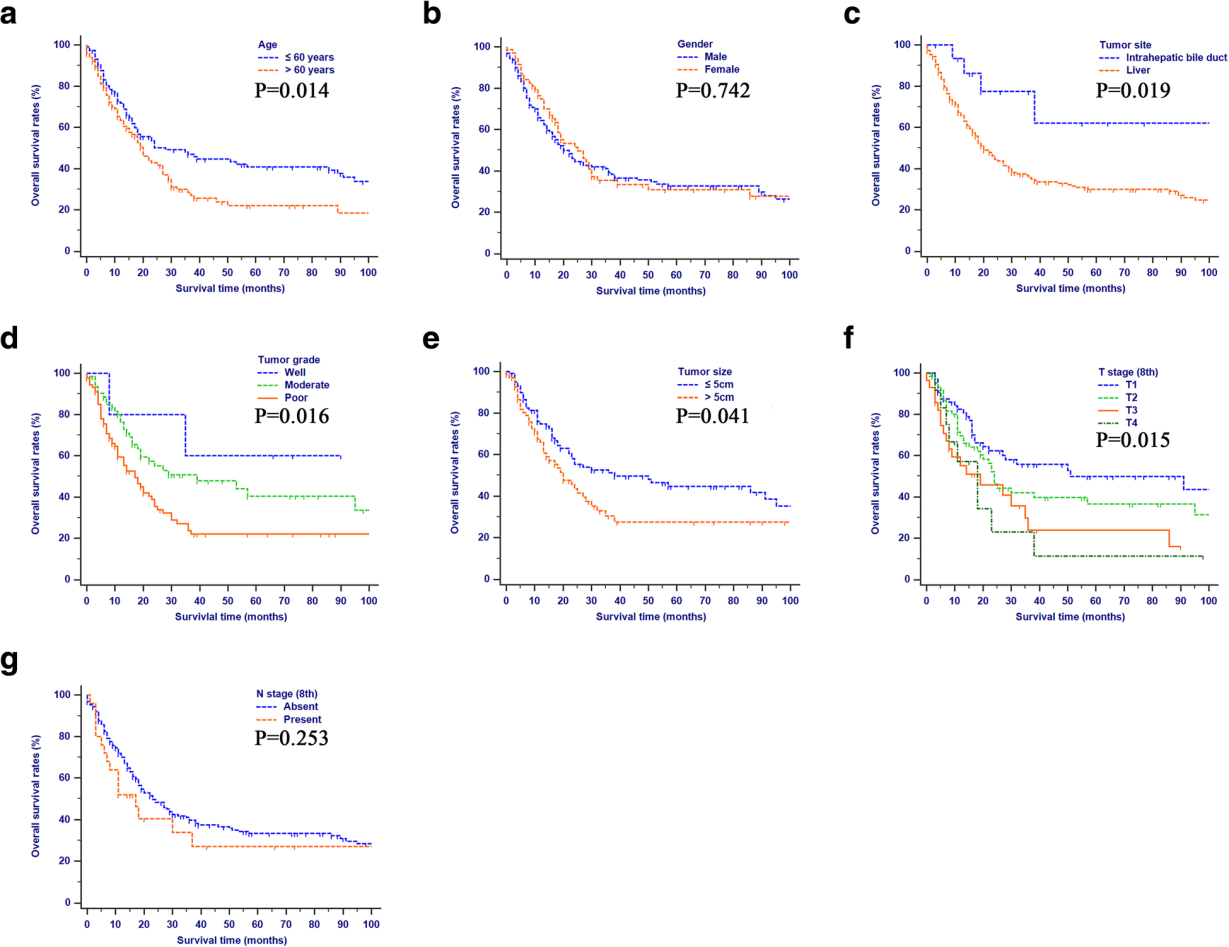
Overall survival rates according to clinical and pathological characteristics: (**a**) Age; (**b**) Gender; (**c**) Tumor site; (**d**) Tumor grade; (**e**) Tumor size; (**f**) T stage (8th); (**g**) N stage (8th). Abbreviations: LN, lymph node

The median OS and CSS for patients were 22.0 (95% CI: 18.0–29.0) months and 27.0 (95%CI: 20.0–37.0) months, respectively. The 1-, 2 and 3-year OS were 67.7, 46.8 and 37.9%, and the 1-, 2 and 3-year CSS were 73.1, 52.0 and 43.0%, respectively. The Kaplan-Meier curves of OS analyses were shown in Fig. 2. Patients who were younger than 60 years old or had smaller tumor (≤ 5 cm) had significant longer OS. Tumor originated from intrahepatic bile ducts, well differentiated tumor, or earlier T stage (8th) also indicated better OS.

### Construction and validation of nomograms

Univariate analyses were performed to filter prognostic factors. It was revealed that age, tumor site, tumor grade, tumor size and T stage (8th) were significantly associated with OS. After a stepwise removal of variables, age (HR = 1.031, 95% CI = 1.006–1.056, *P* = 0.015), tumor grade (HR = 2.049, 95% CI = 1.277–3.288, *P* = 0.003) and T stage (8th) (HR = 1.410, 95% CI = 1.071–1.855, *P* = 0.014) remained significant predictors for OS (Table 3). Proportional subdistribution hazard assumption for CSS analysis also showed that Age (HR = 1.038, 95% CI = 1.010–1.067, *P* = 0.008), tumor grade (HR = 2.027, 95% CI = 1.195–3.439, *P* = 0.009), tumor size (HR = 1.849, 95% CI = 1.001–3.427, *P* = 0.049) and T stage (8th) (HR = 1.429, 95% CI = 1.038–1.969, *P* = 0.029) were all independently associated with CSS.

**Table 3:** Univariate and multivariate analyses of survival in patients with CHCC after surgery

Characteristic		Overall survival						Cancer-specific survival					
		Univariate analysis			Multivariate analysis			Univariate analysis			Multivariate analysis		
		HR	95%CI	P	HR	95%CI	P	HR	95%CI	P	HR	95%CI	P
Age (years)	< 60/≥ 60	1.509	1.080–2.109	0.016	1.031	1.006–1.056	0.015	1.674	1.156–2.424	0.006	1.308	1.010–1.067	0.008
Gender	Male/Female	0.942	0.660–1.346	0.745			NI	1.079	0.734–1.585	0.700			NI
Tumor site	Intrahepatic biliary tract/Liver	3.058	1.131–8.267	0.028	2.081	0.650–6.660	0.217	2.575	0.949–6.981	0.063			NI
Tumor grade	Well/Moderate/Poor	1.738	1.177–2.564	0.005	2.049	1.277–3.288	0.003	1.788	1.155–2.765	0.009	2.027	1.195–3.439	0.009
Tumor size	< 5 cm/≥ 5 cm	1.531	1.010–2.320	0.045	1.337	0.791–2.262	0.278	1.912	1.202–3.043	0.006	1.849	1.001–3.427	0.049
T stage (8th)	T1/T2/T3/T4	1.398	1.130–1.729	0.002	1.410	1.071–1.855	0.014	1.505	1.188–1.905	0.001	1.429	1.038–1.969	0.029
LN metastasis	Absent/Present	1.351	0.800–2.282	0.261			NI	1.192	0.653–2.173	0.568			NI

*CHCC* combined hepatocellular cholangiocarcinoma, *LN* lymph node, *HR* hazard ratio, *CI* confidence interval, *NI* not included

**Fig. 3:**
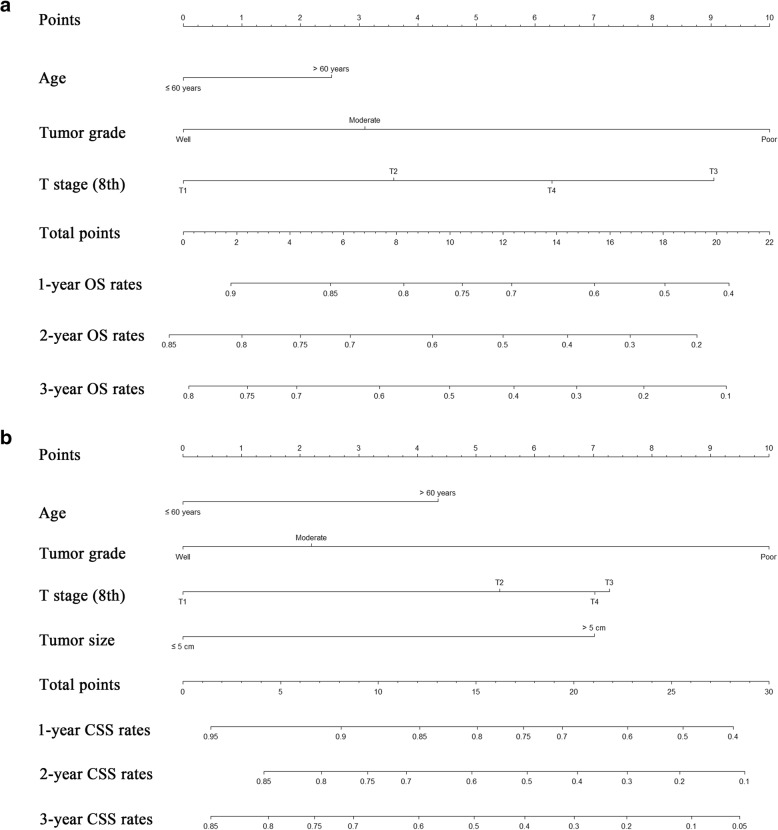
Nomograms predicting 1-, 2- and 3-year OS (**a**) and CSS (**b**) of patients with combined hepatocellular cholangiocarcinoma. Abbreviations: OS, overall survival; CSS, cancer-specific survival

**Fig. 4:**
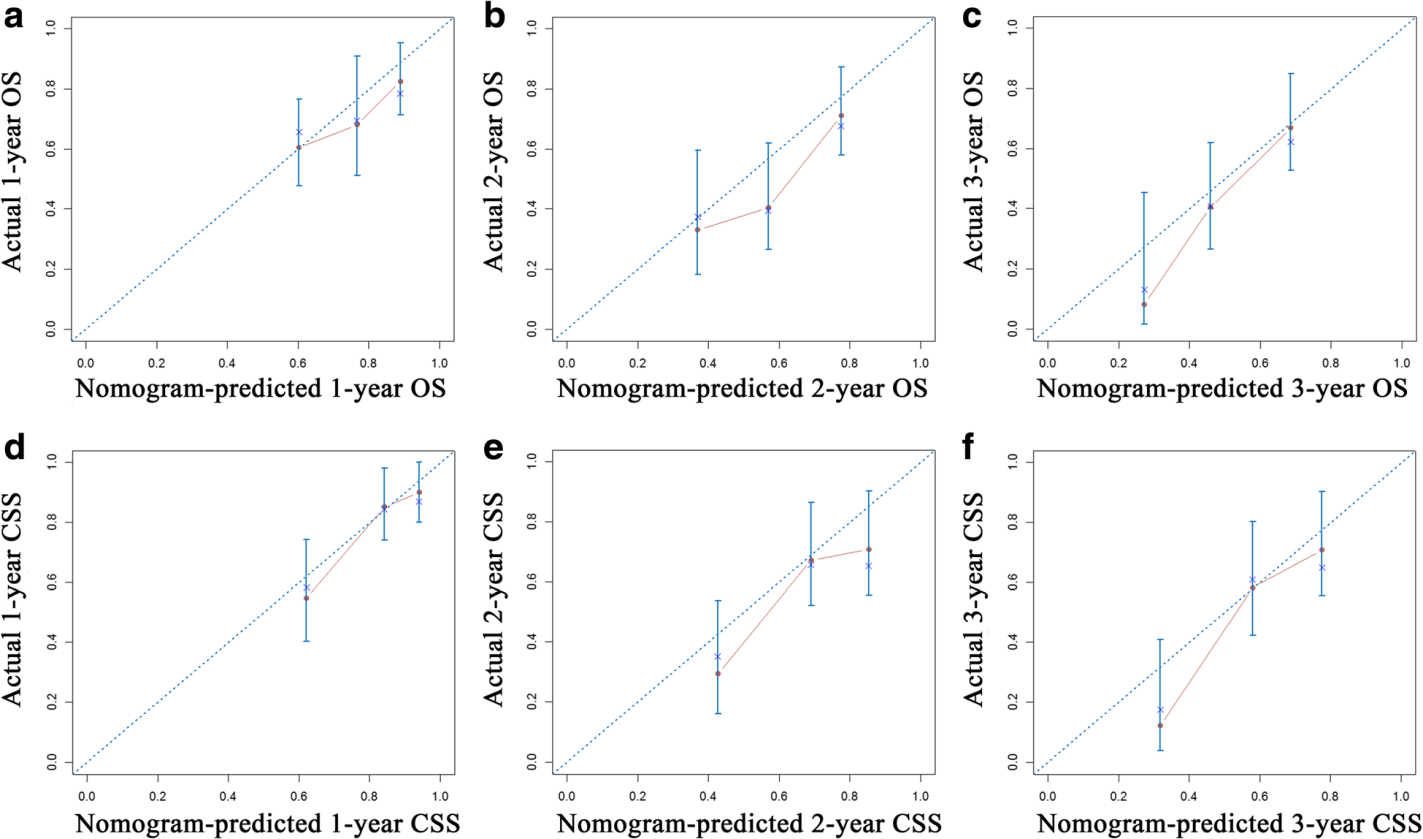
Calibration plots of the nomogram for 1-, 2- and 3-year OS (**a**, **b**, **c**) and CSS (**d**, **e**, **f**) prediction. X-axis represents the nomogram-predicted probability of survival; Y-axis represents the actual OS probability. A perfectly accurate nomogram prediction model would result in a plot that the observed and predicted probabilities for given groups fall along the 45-degree line. Dots with bars represent nomogram-predicted probabilities along with 95% confidence interval. Abbreviations: OS, overall survival; CSS, cancer-specific survival

Nomograms for predicting OS and CSS were constructed with all of the independent predictors of patients in the training cohort (Fig. 3). The C-indexes for OS and CSS prediction were 0.652 (95% CI = 0.579–0.725) and 0.706 (95% CI = 0.630–0.782), respectively, showing good accuracy of the established nomograms for survival prediction. In addition, the comparison of C-indexes of the established nomograms and the 8th edition TNM stage system showed that the established nomograms had enhanced discriminatory ability in predicting OS and CSS (OS, C-index = 0.652, 95%CI = 0.579–0.725 vs C-index = 0.567, 95%CI = 0.492–0.642, *P* = 0.015; CSS, C-index = 0.706, 95%CI = 0.630–0.782 vs C-index = 0.553, 95%CI = 0.469–0.637, *P* < 0.001). The accuracy of nomogram was verified by bootstrapped resamples via the validation cohort. Fair agreement between the nomogram-predicted survival and the actual survival was observed (Fig. 4) and it was indicated that discrimination of nomogram with regard to the SYSUCC validation cohort was also higher than that of 8th edition TNM stage system even though it did not exhibit independent significance (Table 4).

**Table 4:** C-indexes for the nomograms and TNM staging systems in patients with PC after IRE treatment

Survival		Training set	P	Validation set	P
Overall survival	Nomogram	0.652 (0.579–0.725)	Reference	0.659 (0.571–0.747)	Reference
	TNM 8th stage	0.567(0.492–0.642)	0.015	0.592 (0.505–0.679)	0.088
Cancer-specific survival	Nomogram	0.706 (0.630–0.782)	Reference	0.763 (0. 689–0.837)	Reference
	TNM 8th stage	0.553 (0.469–0.637)	< 0.001	0.684 (0.603–0.765)	0.073

Abbreviations as in Table 1

**Fig. 5:**
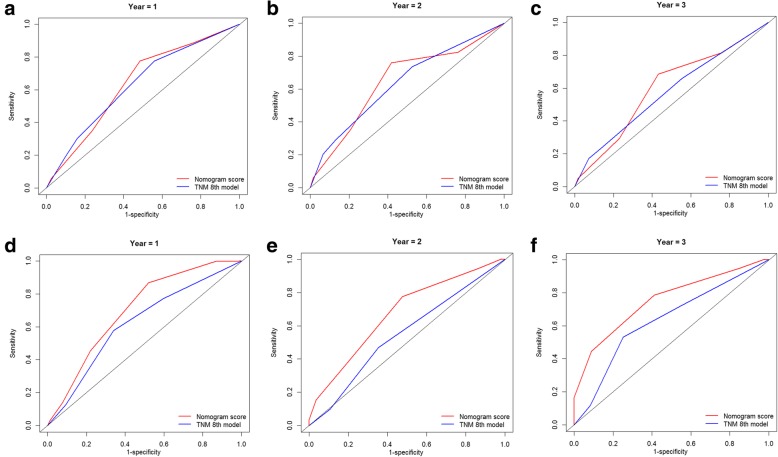
Comparison of the ROC curves of the nomogram and the TNM stage system for 1-, 2- and 3-year OS prediction in the train cohort (**a**, **b**, **c**) and validation cohort (**d**, **e**, **f**). Abbreviations: OS, overall survival; TNM, Tumor-Node-Metastasis

**Fig. 6:**
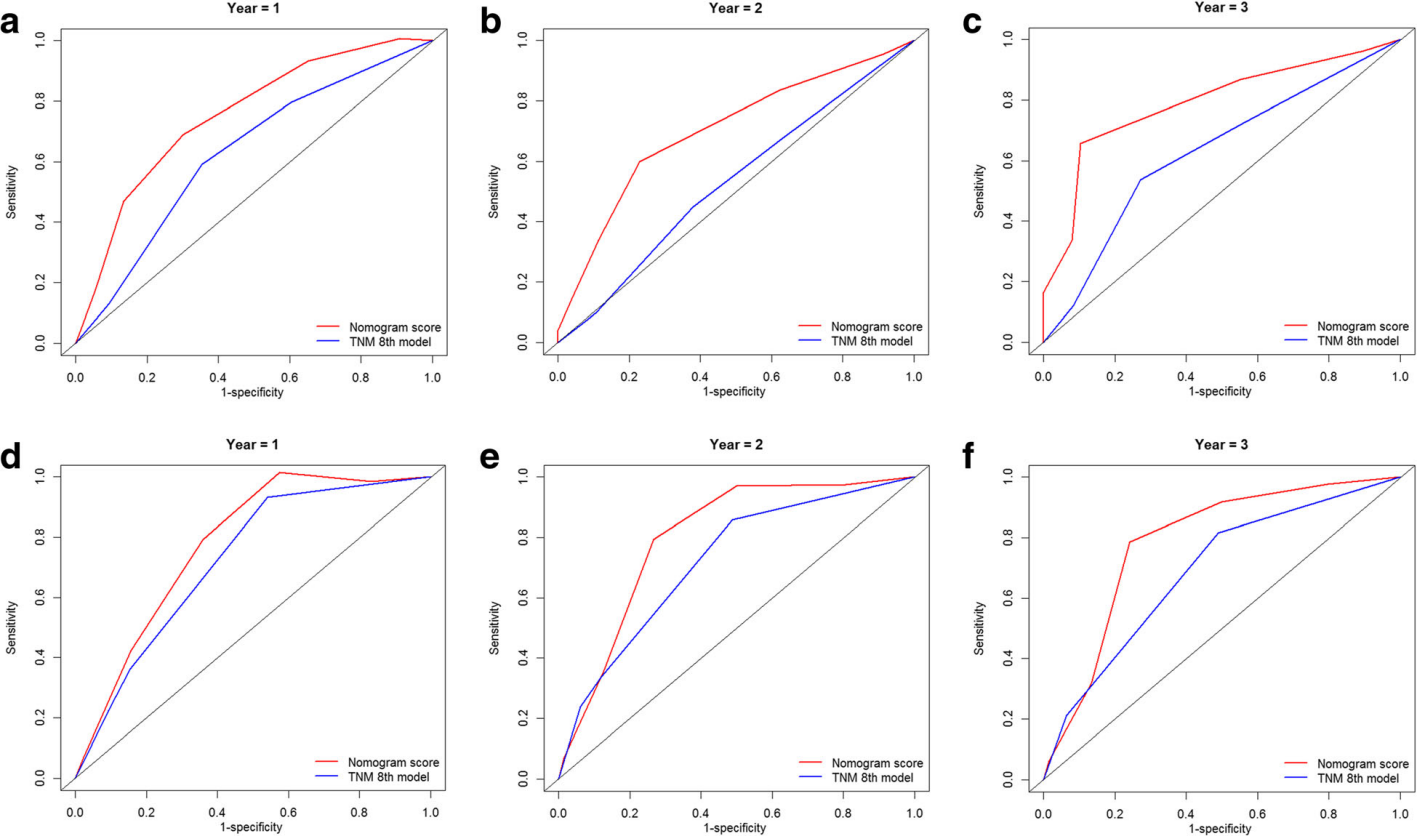
Comparison of the ROC curves of the nomogram and the TNM stage system for 1-, 2- and 3-year CSS prediction in the train cohort (**a**, **b**, **c**) and validation cohort (**d**, **e**, **f**). Abbreviations: CSS, cancer-specific survival; TNM, Tumor-Node-Metastasis

Furthermore, two ROC models of OS and CSS regarding the prediction ability were compared (Table 5). In the training cohort, the values of AUC of the nomogram for predicting 1-, 2 and 3-year OS and CSS were 0.703, 0.675 and 0.753; 0.752, 0.702 and 0.791, respectively, which were all higher than those of 8th edition TNM stage system (Fig. 5). Regarding to the validation cohort, the values of AUC of the nomogram for predicting 1-, 2 and 3-year OS and CSS were 0.638, 0.647 and 0.600; 0.775, 0.800 and 0.785, respectively, whereas the AUC values of the 8th edition TNM stage system for predicting 1-, 2 and 3-year OS and CSS were 0.630, 0.638 and 0.575; 0.722, 0.720 and 0.689, respectively (Fig. 6). The established nomograms showed superior discriminatory capacity than 8th TNM stage system for predicting OS and CSS in both training and validation cohort.

**Table 5:** Values of AUR for the nomograms and TNM staging systems in patients with PC after IRE treatment

Survival		Training set			Validation set		
		1-year	2-year	3-year	1-year	2-year	3-year
Overall survival	Nomogram	0.703	0.675	0.753	0.638	0.647	0.600
	TNM 8th stage	0.623	0.549	0.633	0.630	0.638	0.575
Cancer-specific survival	Nomogram	0.752	0.702	0.791	0.775	0.800	0.785
	TNM 8th stage	0.628	0.530	0.628	0.722	0.720	0.689

*AUC* area under ROC curve; other abbreviations as in Table S1

## Discussion

CHCC is a primary malignant tumor and represents a small proportion of all liver cancers. Due to the rarity of CHCC, most previous studies of CHCC were only limited to single-center cohorts with small sample sizes. The clinicopathological predictors of CHCC remained unclear and the special predictive system was unavailable for the personal treatment. Moreover, most previous studies mainly focused on OS, other than CSS, which reflected the nature of causes of deaths in cancer patients, especially those with increasing ages [19]. Thus, we tried to evaluate the mortality of patients and built nomograms to predict OS and CSS for patients with CHCC after surgery in this study.

It was observed that the increasing ages had a negative effect of survival in patients with CHCC after surgery, which was more obvious on CSS than OS. Moreover, similar with other studies [20, 21], it was indicated that the increasing ages were shown to be independent prognostic factors of survival in this study. Thus, maybe considering age was more appropriate when prognosis of patients with CHCC after surgery was evaluated.

In the presence of competing risk model, other independent prognostic factors included tumor grade, tumor size and T stage (8th). Tumor size is the predominant feature of T stage (8th) and an important component of the 8th edition TNM stage system. It was shown that advanced T stage (8th) represented greater risks of lower OS and CSS in this study. In addition, heavier weight from T stage (8th) in predicting CSS than OS was observed, showing cancer-specific mortalities were more largely depended on inherent feature of tumor. Another factor reflected the intrinsic nature of tumor, tumor grade, was also associated with changes of prognoses of patients with CHCC, which was in accordance with many previous studies [12, 22, 23]. The addition of tumor grade, which was independent of other prognostic factors, such as tumor size and LN metastasis, might contribute to more accurate estimation of tumor behavior and survival outcomes of patients [24].

The differences of origin and the complex nature may lead to the unique features of CHCC compared with HCC and ICC. The predictive significance was not observed for LN metastasis in patients with CHCC in this study. This result was similar with that from a large-scale study [25]. The proportion of patients who were accompanied with LN metastasis was extremely low. In this study, LN metastasis was depended on surgical resection and pathologic confirmation, other than imaging scan. This criterion could contribute to the lower rates of LN metastasis. In addition, similar with other similar studies [23, 25], as an important indicator of advanced TNM stages, LN metastasis was failed to indicate inferior survival in our study, which could partly explain why the loss of monotonous gradient for survival prediction of TNM stage and the superior predictive power of the established nomograms in our study.

With the increasing occurrence and concern of competing risk events, more and more focuses have been paid on competing analyses, such as lung cancer [21], breast cancer [26] and gastric cancer [27]. Considering the non-cancer events contributed to 16.9% of deaths, competing interests were taken into account in survival analyses in this study. As far as we know, it was the first time to build prognostic nomograms to specially predict OS and CSS for patients with CHCC after surgery based on competing risk analysis. Significantly elevated predictive power was observed for the established nomograms in this study. The inclusion of additional variables guaranteed that nomograms were better in predicting OS and CSS, compared with the 8th edition TNM stage system. In addition, the nomograms were established based on a population-based dataset and cross-validated from an external dataset, making our results more generable than those from studies of small cohort or single center. Thus, a diverse range of parameters of CHCC patients are assessed by doctors more objectively and precisely based on the established nomograms. In addition, this newly established system can be used to identify subgroups of patients with a more homogeneous prognosis, estimate individual survival, and then to specialize personal treatment.

There were several limitations for this study. The major limitation of the present study is that not all risk factors were included to construct the nomograms. Some important tumor biomarker, such as carbohydrate antigen 19–9 (CA19–9), and some positive prognostic variables, such as surgical margin status and vascular invasion, were unavailable in SEER dataset. Maybe the additional inclusion of these variables might elevate the predictive power. This is also the major part of our future research. Another limitation is that although the established nomograms showed good discrimination and validation, the values of C-index and AUC are not relatively high. Further validation based on large-scale cohort is needed for these nomograms.

## Conclusion

In conclusion, competing risk analyses were conducted and nomograms specially to predict OS and CSS for these patients were established for the first time in this study. The established nomograms can be used to accurately provide valuable prognostic information, allowing tailed treatments for patients with CHCC after surgery.

## Abbreviations

AUC: Area under ROC curve
CA19–9: Carbohydrate antigen 19–9
CHCC: Combined hepatocellular cholangiocarcinoma
CI: Confidence interval
CIF: Cumulative incidence function
C-index: Concordance index
CSS: Cancer-specific survival
HCC: Hepatocellular carcinoma
HR: Hazard ratio
ICC: Cholangiocarcinoma
LN: Lymph node
OS: Overall survival
ROC: Receiver operating characteristic
SEER: Surveillance, Epidemiology, and End Results
SYSUCC: Sun Yat-Sen University Cancer Center
TNM: Tumor-node-metastasis
